# A Methodology for Remote Microwave Sterilization Applicable to the Coronavirus and Other Pathogens Using Retrodirective Antenna Arrays

**DOI:** 10.1109/JERM.2021.3077110

**Published:** 2021-05-03

**Authors:** Konstantinos Kossenas, Symon K. Podilchak, Davide Comite, Pascual D. Hilario Re, George Goussetis, Sumanth K. Pavuluri, Samantha J. Griffiths, Robert J. Chadwick, Chao Guo, Nico Bruns, Christine Tait-Burkard, Jürgen G. Haas, Marc P.Y. Desmulliez

**Affiliations:** School of Engineering, Institute of Digital CommunicationThe University of Edinburgh 3124 EH8 8AQ Edinburgh U.K.; Department of Information Engineering, Electronics and TelecommunicationsSapienza University of Rome 9311 RM 00185 Roma Italy; Institute of Sensors Signals and Systems, School of Engineering and Physical SciencesHeriot-Watt University 3120 EH14 4AS Edinburgh U.K.; Edinburgh Medical School Infection MedicineThe University of Edinburgh 3124 EH8 8AQ Edinburgh U.K.; Department of Pure and Applied ChemistryUniversity of Strathclyde 3527 G1 1XQ Glasgow U.K.

**Keywords:** COVID-19, medical devices, microwave heating, near field, retrodirective array, SARS-CoV-2, remote sterilization

## Abstract

This paper describes an innovative remote surface sterilization approach applicable to the new coronavirus, severe acute respiratory syndrome coronavirus 2 (SARS-CoV-2). The process is based on the application of a liquid film on the surface or object under sterilization (OUS). A beacon signal is used to self-steer the transmitted power from the designed retrodirective antenna array (RDA) towards the OUS using circularly polarized fields; then, the sterilization is completed by raising and maintaining the required temperature for a certain time. Results suggest that the process takes 5 minutes or less for an angular coverage range over 60 degrees whilst abiding by the relevant safety protocols. This paper also models the power incident onto the OUS, providing consistent results with full-wave simulations. A practical RDA system is developed using a 2 × 1 microstrip patch array operating at 2.5 GHz and tested through the positioning of a representative target surface. Measurements, developed by sampling the power transmitted by the heterodyne RDA, are reported for various distances and angles, operating in the near-field of the system. To further validate the methodology, an additional experiment investigating virus deactivation through microwave heating was also developed. Measurements have been performed with an open cavity microwave oven on the Coronavirus (strain 229E) and egg white protein in a cuvette. This demonstrates that the temperature increases of aqueous films up to 70 $^{\circ }$C by remote microwave-induced heat can denature proteins and deactivate viruses. Possible applications of the method include sterilization of ambulances, medical equipment, and internet of things (IoT) devices.

## I. Introduction and Motivation

The World Health Organization (WHO) has categorized the severe acute respiratory syndrome coronavirus 2 (SARS-CoV-2) among a family of viruses that can affect animals or humans, causing the COVID-19 disease [1]. The unprecedented situation the entire world is facing with COVID-19 calls for new solutions that help to ensure that medical staff, and the general public, are safe and protected. Understanding the way in which the virus propagates and how the infection occurs is of paramount importance to control the pandemic. When people infected with COVID-19 cough or exhale, droplets are expelled from their nose or mouth and they can diffuse onto objects and surfaces [1]. The virus can survive for hours, or even up to days, on plastic and metallic surfaces [1], which means that people can inadvertently become infected with COVID-19, becoming potential carriers. These emitted droplets, depending on their size and weight, can also remain suspended in air. The survival of the virus on contaminated surfaces has a vital role in its spreading, therefore sterilization of said surfaces is important to curtail the pandemic.

Accurate information regarding the life-time of the SARS-CoV-2 on infected surfaces is still not completely clear. Based on some early results [1] it is expected to act like other coronaviruses. A preliminary work developed in 2003, when the SARS-CoV-1 was discovered, reported on the stability of coronaviruses in human specimens and related environments. The results made it clear that for at least 96 hours SARS was capable of surviving and being infectious in serum, diluted sputum and feces, and up to 72 hours for urine. At the same time the virus can remain strongly infectious at low and room temperatures for at least 2 hours. Only with longer exposure times and at higher temperatures did the virus start to become non-infectious. In addition, ultraviolet C (UVC) irradiation for 9 minutes has been shown to lead to the elimination of the virus [2].

Recent research has also shown that viruses with pandemic potential, like SARS, have a long survival time on dry surfaces and can infect field settings [3]. Coronaviruses can also survive in water and pre-pasteurized settled sewage, making human exposure possible [4]. Aerosol and surface stability of the new SARS coronavirus has also been recently studied in [5] using different materials: copper, cardboard, stainless steel, and plastic. The concentration of the virus (i.e., the titer) was measured at 21 $^{\circ }$C to 23 $^{\circ }$C and 40% relative humidity for more than 7 days. The titer of the aerosolized virus was expressed in 50% tissue-culture infectious dose (TCID50) per liter of air. The experiments were repeated three times each. Table I presents the titer results, showing that on cardboard and stainless steel it becomes very low after 24 hours. In aerosol form and on copper, the titers require only a few hours to drop to low levels. However, the virus durability on plastic can be very long (up to 48 hours) [5].

**TABLE I:** Survival of Coronavirus in Aerosols and on surfaces [5]

Titer	Hours of Viability of Virus
Aerosols	$< $ 3
Copper	8
Plastic	72
Cardboard	24
Stainless Steel	$< $ 48

Abraham and colleagues recently developed guidelines for microwave thermal sterilization in [6]. The authors suggest that a temperature beyond 60 $^{\circ }$C is required for the thermal destruction of the virus; i.e. SARS-CoV-2, highlighting that the time needed to complete the sterilization is a function of temperature. Specifically, a 3 minute exposure at 75 $^{\circ }$C is recommended, whereas an exposure of 5 minutes at 65 $^{\circ }$C and of 20 minutes at 60 $^{\circ }$C is recommended, adopting a conservative approach on the sterilization time and temperature. Additionally, the Centers for Disease Control and Prevention (CDC) in the United States has published a guideline for disinfection and sterilization methods in health-care facilities, including alcohol, chlorine, and pasteurization [7]. Sterilization can be operated through ionizing and infrared radiation (indicating powers in the order of 600 W [8]), which are well-established but are relatively expensive methods, often requiring bulky systems. These features not always match the requirements of health-care facilities (ex. US Food and Drug Administration, FDA) [7]. Microwaves are also regarded as a promising method for sterilization and have a number of applications, which include soft contact lenses, dental instruments, dentures, milk, urinary catheters and many others [7].

The general principle commonly used in a kitchen-based microwave oven can be exploited to produce heat for sterilization [7]. Basically, microwaves can generate friction of the constituent water molecules, usually at 2.45 GHz; typically, an effective microbicide dose requires 60 seconds to 5 minutes, depending on the microorganism being targeted [7]. During radiation, the electric field hits the polar molecules, generating vibrations as well as twisting, turning, and stretching, which causes friction in a viscous environment, thus creating heat. The mechanism is affected by the amount of water present and by the intensity of the incident field [9].

UV radiation is also a natural disinfectant that reduces health-care acquired infections and kills pathogens. It is typically applied to sterilize drinking water and air [10]. There are various factors such as temperature, wavelength, and intensity, which influence the effectiveness of the sterilization process. From a physical viewpoint, UV radiation is associated with wavelengths between 100 and 400 nm, while maximum bactericidal effect takes place at 240 to 280 nm [10]. In health-care environments, UV covers the inactivation of airborne organisms and microorganisms on surfaces [7]. UV can be applied in health-care facilities or emergency vehicles, such as ambulances, which are disinfected by using UV light. Conventional decontamination time can take about 15–20 minutes [10].

Research exploring the possibility of eliminating viruses based on a method using confined acoustic vibrations (CAV) was published in [11]. In this approach, energy can be transferred by an incident microwave power density by CAV, which induces stress fractures in the virus structure. This operation has shown to cause deactivation of the airborne virus, whilst being safe for the public. Moreover, an experiment took place to understand the power that was needed for the destruction of the virus, using a horn antenna, with the viral samples being placed below it. The results showed that between 6 to 12 GHz was necessary for the destruction of the viruses with input powers from 0.57 to 3.92 W [11].

We propose here a new methodology and procedure for the remote microwave thermal sterilization of infected surfaces using antennas and electromagnetic (EM) waves at microwave frequencies. The technique is modeled and developed to act against the SARS-CoV-2 causing the COVID-19 disease. The technique can be used in various medical scenarios, such as hospital rooms or ambulances, for contact-less sterilization and virus deactivation. The level of required energy and the possible automation of the system in particular, clearly differentiates the proposed method from other sterilization approaches. For example, only a few Watts are required, offering remote operation with RF beam focusing, not requiring the operator to come in contact with the contaminated surface. In contrast to a more conventional disinfectant spray, this approach is not targeted and focused; i.e., it typically generates a cone of liquid vapour that disperses in air and further requires manual wiping and cleaning by an operator. In fact, a harsh disinfectant would need to be wiped by an operator, which is not always possible, time efficient or recommended.

The proposed methodology is based on self-steering or retrodirective antenna arrays (RDAs) [12]-[14], which have recently been shown to be advantageous for wireless power transmission systems in the remote charging of batteries within smart phones, tablets, and other devices [15], [16]. There are two basic types of RDAs: passive and active. In the former, the frequencies of the beacon signal and of the re-transmitted signal are typically the same, whereas in active RDAs the two signals can have different frequencies to minimize unwanted electromagnetic coupling. Active RDAs operate by signal heterodyning and are typically referred to as Pon RDAs [12]. Other applications for RDAs include [13], [14] wireless communications, remote sensing, and imaging.

**Fig. 1.**
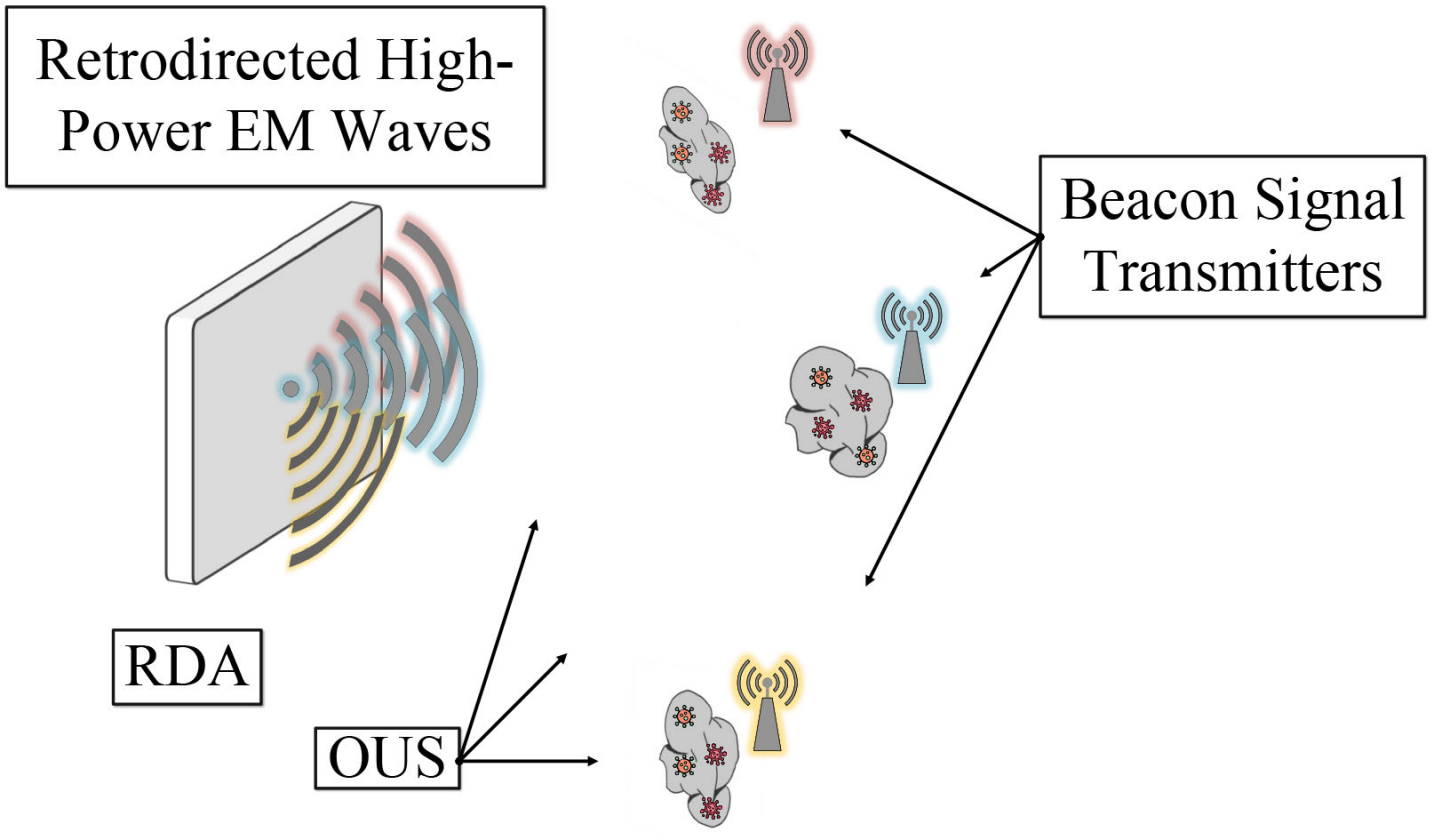
System overview of an RDA operating as a remote microwave thermal sterilizer. The beacon signal transmitter allows for localizing the OUS and the RDA re-radiates power towards that direction.

**Fig. 2.**
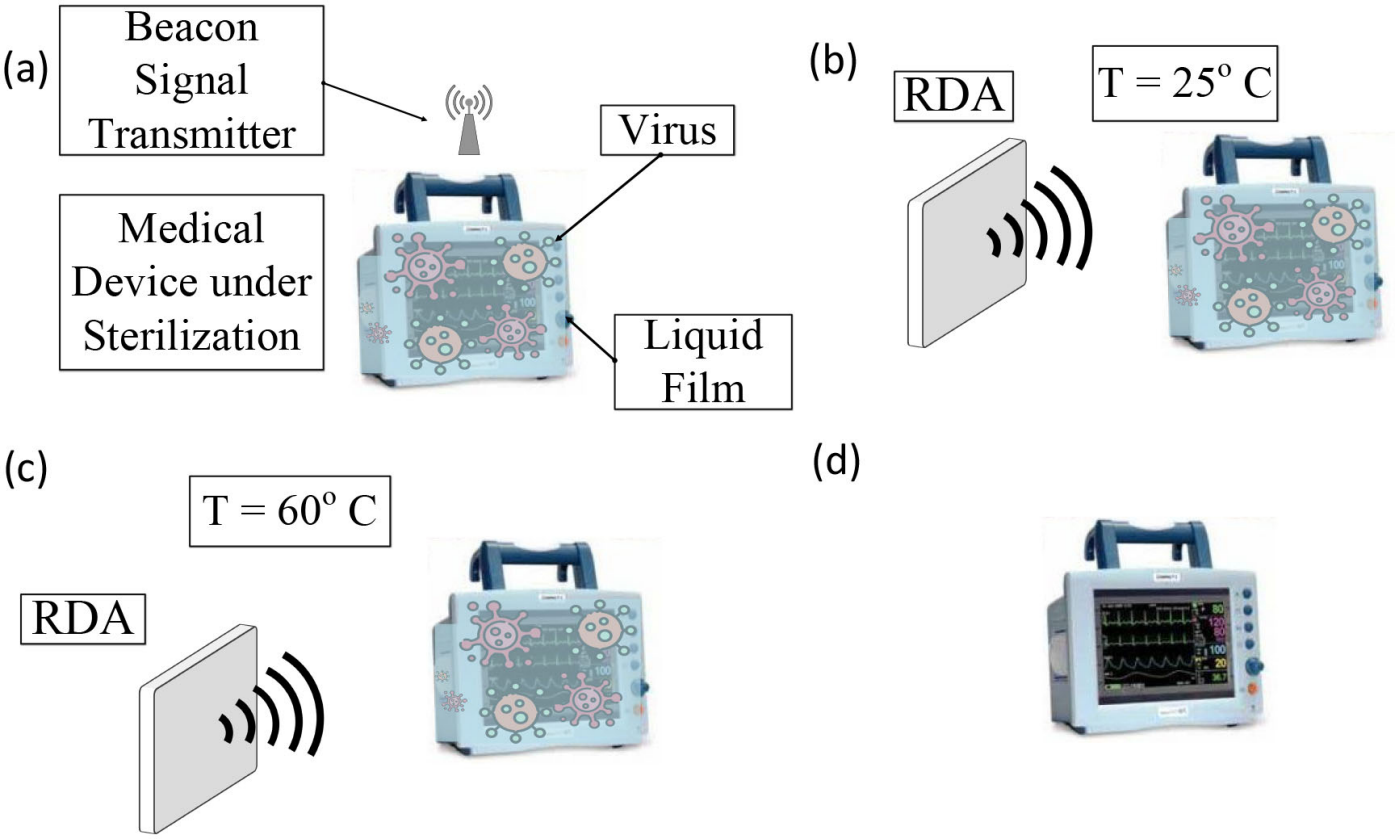
Illustration of the microwave thermal sterilization process for a medical device where a liquid film has been applied on it: (a) The medical device sends a beacon signal towards the RDA, (b) RDA receives the beacon signal and starts to transmit power towards to the medical device, (c) the required temperature of the liquid film has been achieved, and (d) the microwave thermal sterilization has been completed.

The origin point of the beacon signal can be automatically tracked by the RDA without *a priori* knowledge of its physical position. Therefore, an RDA is capable of transmitting a signal back to the beacon location automatically. The main concept of our proposed approach is that the object under sterilization (OUS) has a thin water film and a small antenna on it. The antenna generates the radio beacon (see Figs. 1 and 2), allowing the RDA to localize the OUS, to steer its beam towards it, and to start to transfer power wirelessly for remote microwave thermal sterilization and prompt pathogen deactivation.

Existing WiFi transmitters for data communications could be exploited as the beacon signal [15], [16]. This is because the OUS could be medical devices, tablets, and other IoTs, which might connect to conventional wireless local area networks (whose connection is based on beacon signals) within hospitals, ambulances, and other care facilities for example. If needed, small beacon transmitters could be anyway placed on the required surfaces by operators, enabling hands-free sterilization. This can have significant benefits as the radiated beam can be steered automatically towards the contaminated surface, whilst the operator need not to physically touch it. Furthermore, the proposed RDA sterilization approach could be positioned within the walls of ambulances, for example, and in proximity of other indoor facilities making the system completely autonomous. It could also be made handheld for operators with a given steered beam or fixed to be perpendicular (or broadside) to the sterilizing antenna unit which generates EM waves.

The paper is divided into three parts. The first part deals with the theoretical approach and modeling of the problem. Firstly, the microwave thermal sterilization process using a Pon RDA is analytically described by modeling a medical device as a rectangular surface, which is then sprayed with a thin film of liquid water. This is treated as the OUS in Section II. The required energy for the sterilization process is examined through simulations and numerical calculations using a commercial full-wave EM solver. As the system operates in the near-field (NF), conventional physical quantities, like the radar cross section (RCS) and the power transferred by the RDA are evaluated.

In the second part, the operation of the RDA is simulated and experimentally verified. As outlined in Section III, the OUS is represented by a rectangular surface comprised of two patch antennas operating at two distinct frequencies (2.4 GHz and 2.5 GHz). One acts as the beacon while the other as the effective aperture surface, as required to physically measure the retrodirected high-power signal by the Pon RDA. A parametric analysis has also been developed for some variable distances and angles. The theoretical results of the first section are then compared with simulations and measurements. The power radiated at 2.4 GHz is set at a level capable of achieving the required temperature to start the sterilization, abiding by the relevant international safety protocols.

In the third part, virus inactivation was investigated using an open-end microwave cavity as described in [17]. To experimentally study the inactivation, the human coronavirus strain 229E with a green fluorescent protein (GFP) tag (CoV-229E-GFP) was used [18]. After microwave exposure, residual infectivity was determined by infection of virus-susceptible Huh7 cells and measurement of the replication growth curves. Results are presented in Section IV. Finally, it is demonstrated that microwave induced heating of liquid films to 60 $^{\circ }$C with an open end microwave cavity can lead to protein denaturation, as visually observed for a solution of egg white, and deactivation of the CoV-229E-GFP strain, as demonstrated by the aforementioned experiment. A short discussion and conclusion follows.

## II. Microwave Thermal Sterilization Technique

This section describes the microwave thermal sterilization technique of the OUS by using an RDA and the beacon signal. Classic thermodynamic theory, in combination with conventional wireless power transfer (WPT) and radar techniques, are considered. A test case with an RDA radiating towards a rectangular plate, sprayed with a water layer (assumed as the OUS), from different distances and angles is described. A numerical model of the time required to start the microwave thermal sterilization is developed.

### A. Description of the Microwave Thermal Sterilization System

The system is illustrated in Fig. 1, where the RDA system emits the desired level of microwave power needed to start the microwave thermal sterilization.

The microwave thermal sterilization process is detailed in Fig. 2: a) the contaminated heart rate monitor (or any generic medical device, equipment, etc.) is covered by a liquid film at room temperature (25 $^{\circ }$C), b) the medical device sends the beacon radio signal though a small integrated antenna, c) the RDA receives it and, because of the phase conjugation mechanism made possible by the Pon heterodyne circuit system [12], it steers and radiates power back towards the medical device for a certain temporal interval. As discussed in [6], the virus is deactivated at a minimum temperature of 60 $^{\circ }$C. The required amount of power and time needed for the liquid film to reach 60 $^{\circ }$C are discussed in the following.

**Fig. 3.**
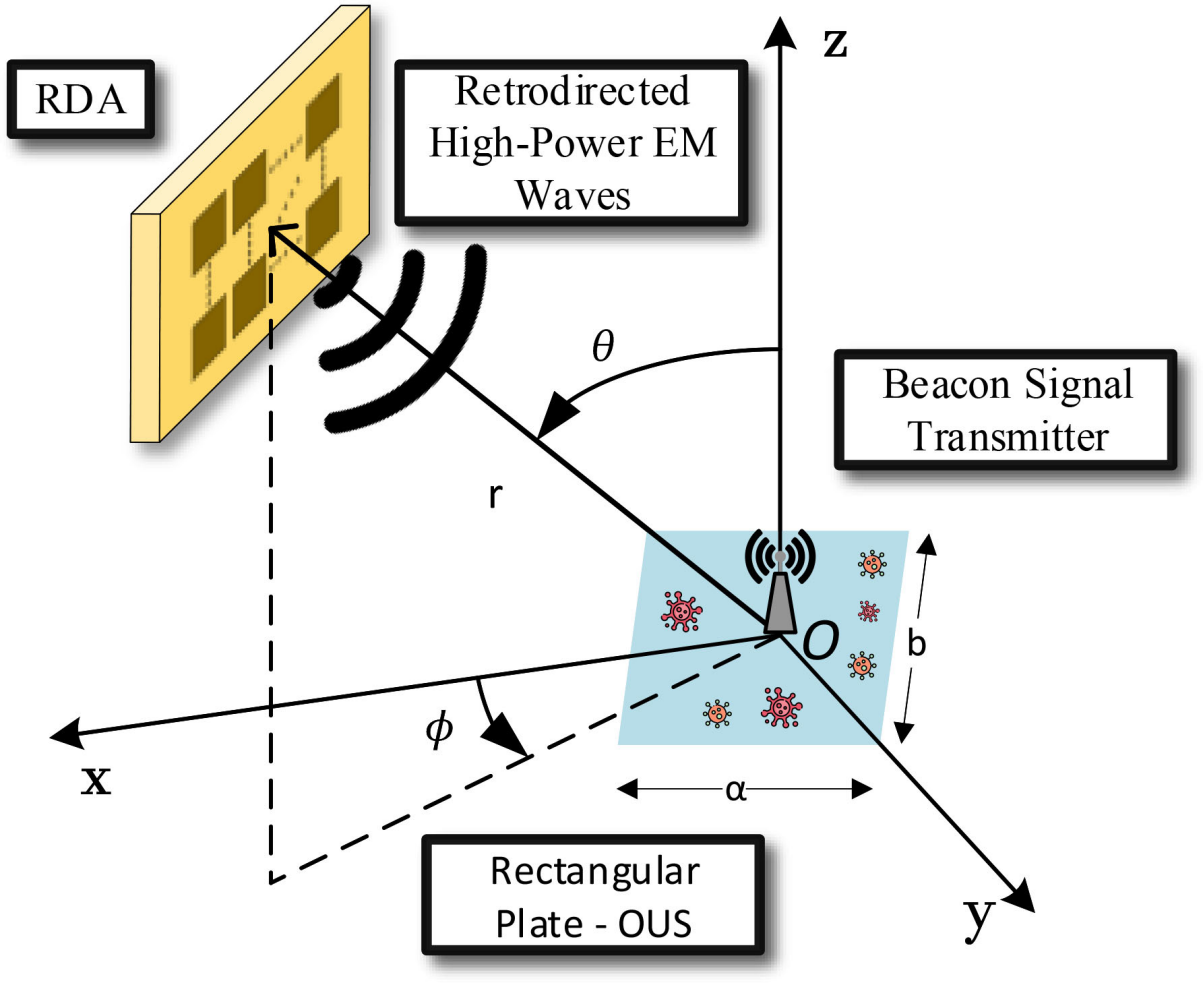
Orientation of the RDA with respect to a rectangular plate (OUS). The beacon signal transmitter allows for localizing the OUS and the RDA transmits power in the NF by an antenna array made by $N_t$ elements towards that direction.

Given the electrically large aperture size for the RDA required to achieve the desired incident power on the OUS positioned at a distance $r$ and the relevant path losses, it expected that the RDA should operate in the NF. The boundary between the NF and the far-field (FF) operation is conventionally approximated by $r = \frac{2D^2}{\lambda }$, where $D$ is the maximum linear dimension of the antenna and $\lambda$ is the wavelength in free-space [19]. Given the RDA transmitted power (i.e., $P_t$) and its gain (i.e., $G_t$), it is possible to calculate the power impinging on the OUS. Indicating with $\theta$ and $\phi$, the elevation incidence angle and the azimuthal incidence angle towards the RDA (Fig. 3), the incident power is given by

\begin{align*}
P_i = & \frac{P_t G_t \left(\theta, \phi \right)}{4\pi r^2} \sigma \left(\theta,\phi \right), \tag{1}
\end{align*}

where $\sigma$ is the target radar cross section (RCS) [20], [21].

As a test case, we consider here a flat rectangular plate OUS with dimensions $\alpha$, $b$. The coordinates of the source position are $(r \, \mathrm{cos}\phi \, \mathrm{sin}\theta, r \, \mathrm{sin}\phi \, \mathrm{sin}\theta, r \, \mathrm{cos}\theta \,)$ where $r = \parallel \mathbf {r}\parallel$. A simple formula for the RCS of a rectangular plate in the NF is given in the following [22]:

\begin{align*}
\sigma _{\mathrm{plate}} \left(\theta,\phi \right) = & \left. \frac{\pi r^2 \mathrm{cos}^2\theta }{4\left(1-c^2\right)\left(1-s^2\right)} \right| F_{1c}F_{1s} \\
& + \frac{3c^2}{1-c^2} F_{1s} \left(F_{1c} + E_{1c} \right) \\
& \left. + \frac{\text{3}\;s^2}{1-s^2} F_{1c} \left(F_{1s} + E_{1s} \right) \right|^2. \tag{2}
\end{align*}

where the Fresnel integral is defined as $F(x) = \int _{0}^{x} e^{\frac{j \pi t^2}{2}} \mathrm{d}t$ and the following definitions holds: $c = \mathrm{sin} \theta \, \mathrm{cos} \phi$, $s = \mathrm{sin} \theta \, \mathrm{sin} \phi$. On this basis, the $F_{1c}$, $F_{1s}$, $E_{1c}$ and $E_{1s}$ functions are given by

\begin{align*}
&{\begin{cases}F_{1c} = F\left(u_c + \upsilon _c \right) + F\left(u_c - \upsilon _c \right) \\
F_{1s} = F\left(u_s + \upsilon _s \right) + F\left(u_s - \upsilon _s \right) \end{cases}} {\kern-14.22636pt}\,\,\, \tag{3} \\
&{\begin{cases}E_{1c} = \frac{1}{j\pi \upsilon _c} \left[ e^{\frac{j\pi (u_c - \upsilon _c)^2}{2}} - e^{\frac{j\pi (u_c + \upsilon _c)^2}{2}} \right] \\
E_{1s} = \frac{1}{j\pi \upsilon _s} \left[ e^{\frac{j\pi (u_s - \upsilon _s)^2}{2}} - e^{\frac{j\pi (u_s + \upsilon _s)^2}{2}} \right] \end{cases}} {\kern-14.22636pt}\, \, \, \tag{4}
\end{align*}

where

\begin{align*}
{\begin{cases}u_c \pm \upsilon _c = \sqrt{\frac{r}{\lambda (1-c^2)}} \left[\frac{\alpha (1-c^2)}{r} \pm 2c \right]\\
u_s \pm \upsilon _s = \sqrt{\frac{r}{\lambda (1-s^2)}} \left[\frac{b (1-s^2)}{r} \pm \text{2}\;s \right] \end{cases}} {\kern-14.22636pt}\,\,\, . \tag{5}
\end{align*}

### B. Simulation of the Radiated Power

**Fig. 4.**
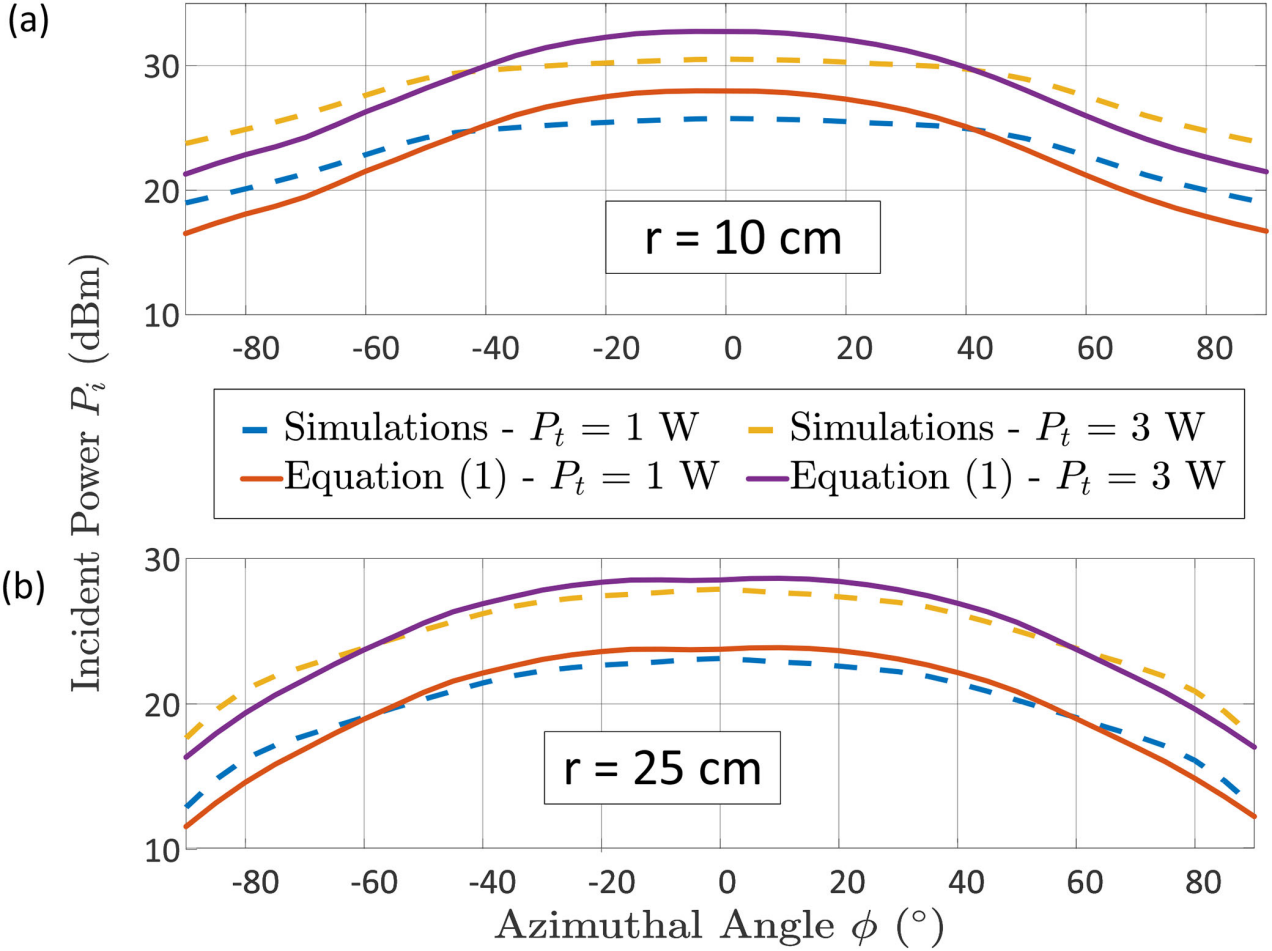
Incident power generated by a 2×1 RDA, for two different input power levels (i.e., 1 W and 3 W); upon on a rectangular water-based aperture considering distances (a) $r$ = 10 cm and (b) $r$ = 25 cm.

We evaluate here the incident power given by (1) performing a simulation. The RDA system is composed of a two-element array operating at 2.5 GHz with input power $P_t$ = 1 W (3 W also considered for comparison); a rectangular water layer (i.e, the OUS) with dimensions $\alpha$ = 14 cm, $b$ = 8 cm, thickness $t$ = 0.25 mm is included in the simulation. The OUS is rotating from -90 $^{\circ }$ to 90 $^{\circ }$ along the azimuthal angle ($\phi$), keeping constant the elevation angle ($\theta$). In each rotation the RDA beam was steered towards the OUS direction and the power incident on it was recorded, evaluating the antenna gain ($G_t (0, \phi)$) is at the relevant direction by (1). The plots in Fig. 4 show the results for two different distances, for $r$ = 10 cm and $r$ = 25 cm; in both cases the RDA operates within the NF.

### C. Required Energy for Microwave Thermal Sterilization

The microwave thermal sterilization of an object requires to control the involved physical parameters, such as the required thermal energy, the temporal duration, and the dimensions of the surface under sterilization. An object with a given mass and heat capacity can be described with the specific heat $c_p$ [23], defined as the ratio between mass and capacity [24]. Thus, the energy stored by the substance can be calculated as $\mathrm{E} = c_p \mathrm{\Delta T} m$ [25], where $E$ is the required heat energy for increasing the temperature of the substance. $\mathrm{\Delta T}$ is the differential temperature between the initial and the final states of the substance and $m$ is the mass of the substance, which can be expressed in terms of the volume ($V$) and the density ($\rho$) of the substance as $m = V {\kern1.42271pt} \rho$ [26]. Assuming that a source transfers thermal energy, the difference in temperature of the substance is equal to $\mathrm{\Delta T}$. In a time interval $t_{\mathrm{total}}$, therefore, the needed power is given by $P = {E}\big /{t_{\mathrm{total}}}$ [27].

Combining (1) with the aforementioned basic energy equations a numerical model of the time needed to increase the temperature is developed. Given the OUS volume, density and heat capacity, the original and desired final temperature, RCS, RDA gain as well as the distance between the two devices, the following expression can retrieved:

\begin{align*}
t_{\mathrm{total}} = & \frac{4\pi r^2}{P_t} \frac{c_p \mathrm{\Delta T} V \rho }{\sigma \left(\theta,\phi \right) G_t \left(\theta, \phi \right)}. \tag{6}
\end{align*}

**Fig. 5.**
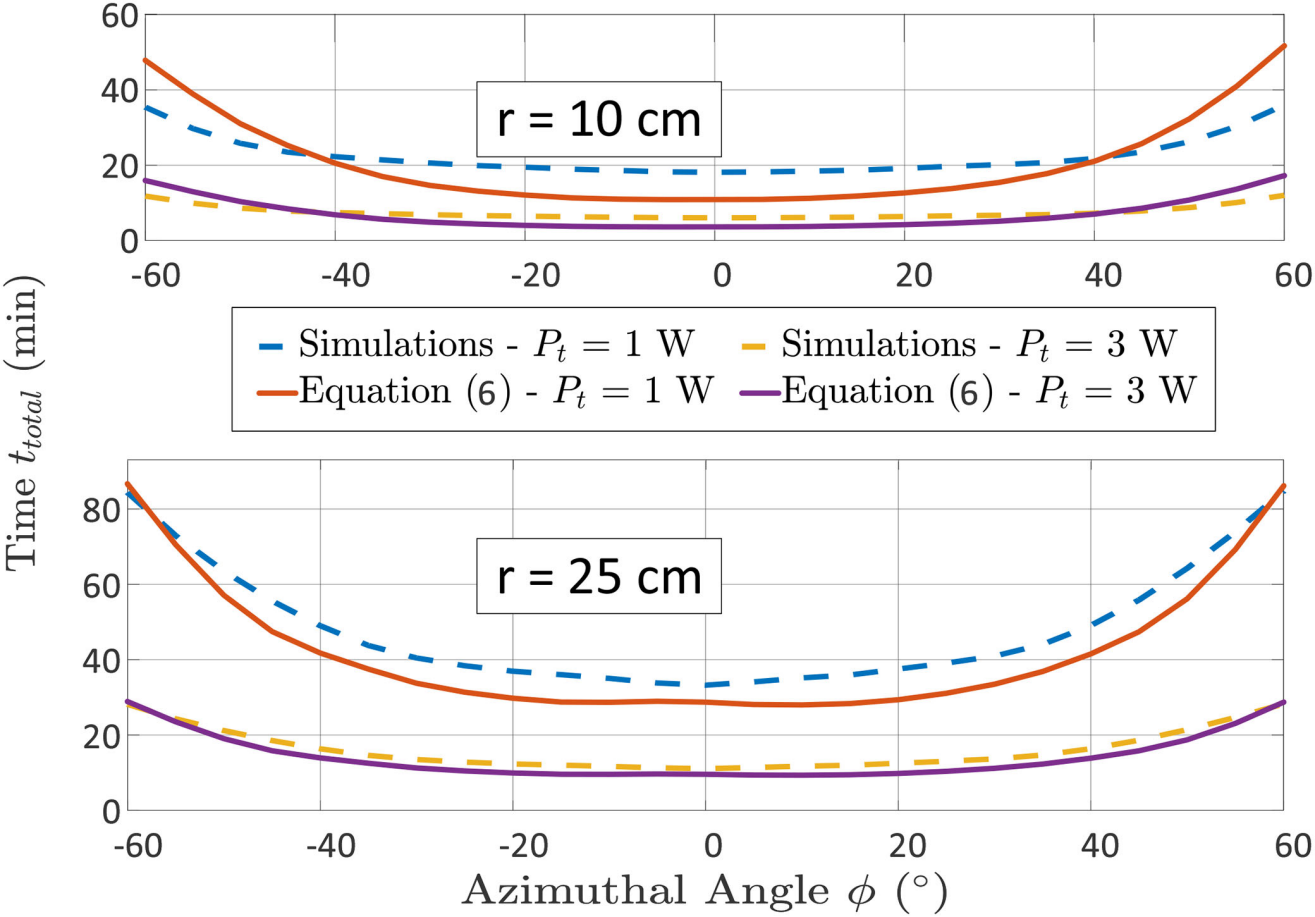
Required time to heat the water film to 60 $^{\circ }$C to induce the sterilization of the coronavirus. Water layer aperture as in Fig. 4. For $\pm$30 $^{\circ }$ azimuthal angles the time is less than 5 min, for an input power of 3 W. It is expected that with increased levels of power, sterilization time can decrease.

The formula in (6) is considered here to evaluate the performance of the system constituted by a 2×1 RDA and the rectangular water layer. The desired temperature was set to 60 $^{\circ }$C, whilst the original temperature was assumed equal to 25 $^{\circ }$C. The specific heat capacity of water is $c_p = 4179.6\;{\mathrm J.kg^{-1}.K^{-1}}$, its density is $\rho = {10^3}\;{\mathrm Kg/m^3}$, and its volume is $V = \alpha \;b\;t\;{\mathrm m^3}$ [28]. Fig. 5 presents the total time needed for the sterilization of water in terms of various distances and azimuthal angles with respect to the RDA, setting a transmitted power $P_t$ = 1 W and 3 W. The results in Fig. 5 demonstrate that the azimuthal angles between the RDA and the OUS required to reach the desired temperature to start the microwave thermal sterilization has a significant impact on the time. As expected, the minimum value is achieved at broadside, (i.e.; $\theta$ = 0 $^{\circ }$). This is because the antenna gain decreases off broadside, highlighting the importance of the self-steering RDA. It should be mentioned that higher input power ($P_t$) and directivity can help to reduce the sterilization time.

**Fig. 6.**
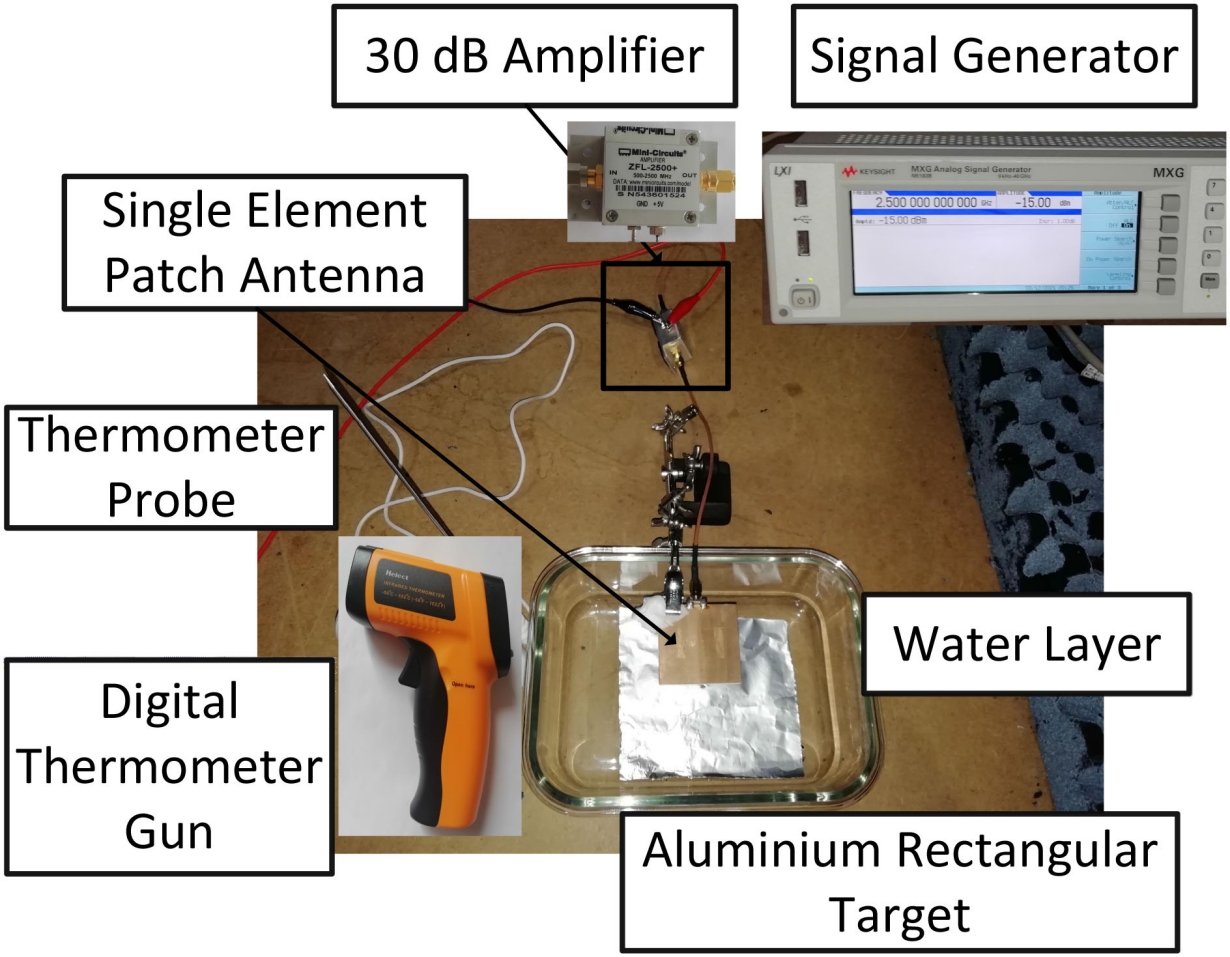
Experimental setup: the relative position between target and the single-element patch antenna was set to 10 cm; incident angle equal to 0 $^{\circ }$.

In order to validate the formula in (6) the time required for raising the temperature by 1 $^{\circ }$C was assessed through a simple experimental setup. This included a single-element patch antenna (linearly polarized) operating at 2.5 GHz and a rectangular aluminium ($\alpha \times \alpha$) target covered by a water layer ($t \approx$ 0.5 cm), placed within an anechoic chamber (Fig. 6). A signal generator (Keysight MXG N5183B) was used with output power equal to $-$15 dBm, connected in series with a 30 dB power amplifier (MiniCircuits ZFL-2500+) so that the input power at the antenna was $P_t$ = 15 dBm (i.e., $\approx$ 0.03 W). The relative distance between the antenna and the target was set to 10 cm and the incident angle at 0 $^{\circ }$. Based on these values and the estimated required time for the changing of temperature by 1 $^{\circ }$C is about 3.5 hours. The original temperature of the target was measured to be 20.3 $^{\circ }$C through a digital laser temperature gun. After 3.5 hours the measured temperature was 21.4 $^{\circ }$C. The temperature around the water layer was also monitored with a digital thermometer equipped by a probe; this temperature was 18.6 $^{\circ }$C at the beginning of the experiment and 19.4 $^{\circ }$C at the final target temperature making these results consistent with (6).

## III. Near-Field RDA

### A. Pon-Based RDA Design and Operation

The Pon RDA is a heterodyne system that avoids the use of circulators since the transmit and receive arrays are isolated; the structure uses RF mixers to provide retrodirectivity by phase conjugation [14]. This principle is simple and well established, it exploits the product between two input signal tones [12], which constitutes the RF input signal and the local oscillator signal (LO). Gain blocks (namely, amplifiers) are typically employed to boost the retrodirected power. The Pon RDA designed in this work follows [15] and collects the beacon signal with a receiving array (with one polarization) and it retrodirects the radiation back to the OUS with a high-power transmitting array with another polarization. The radiating element in this RDA architecture is a two-element circularly polarized (CP) patch array. CP operation is needed to avoid potential polarization mismatches between the target and the RDA while also allowing for orientation free movement of the OUS. Element decoupling strategies were not considered for simplicity in our work, as element coupling values were −20 dB or less. Different approaches, such as in [29], [30], can be adapted to our design if better performance is needed.

**Fig. 7.**
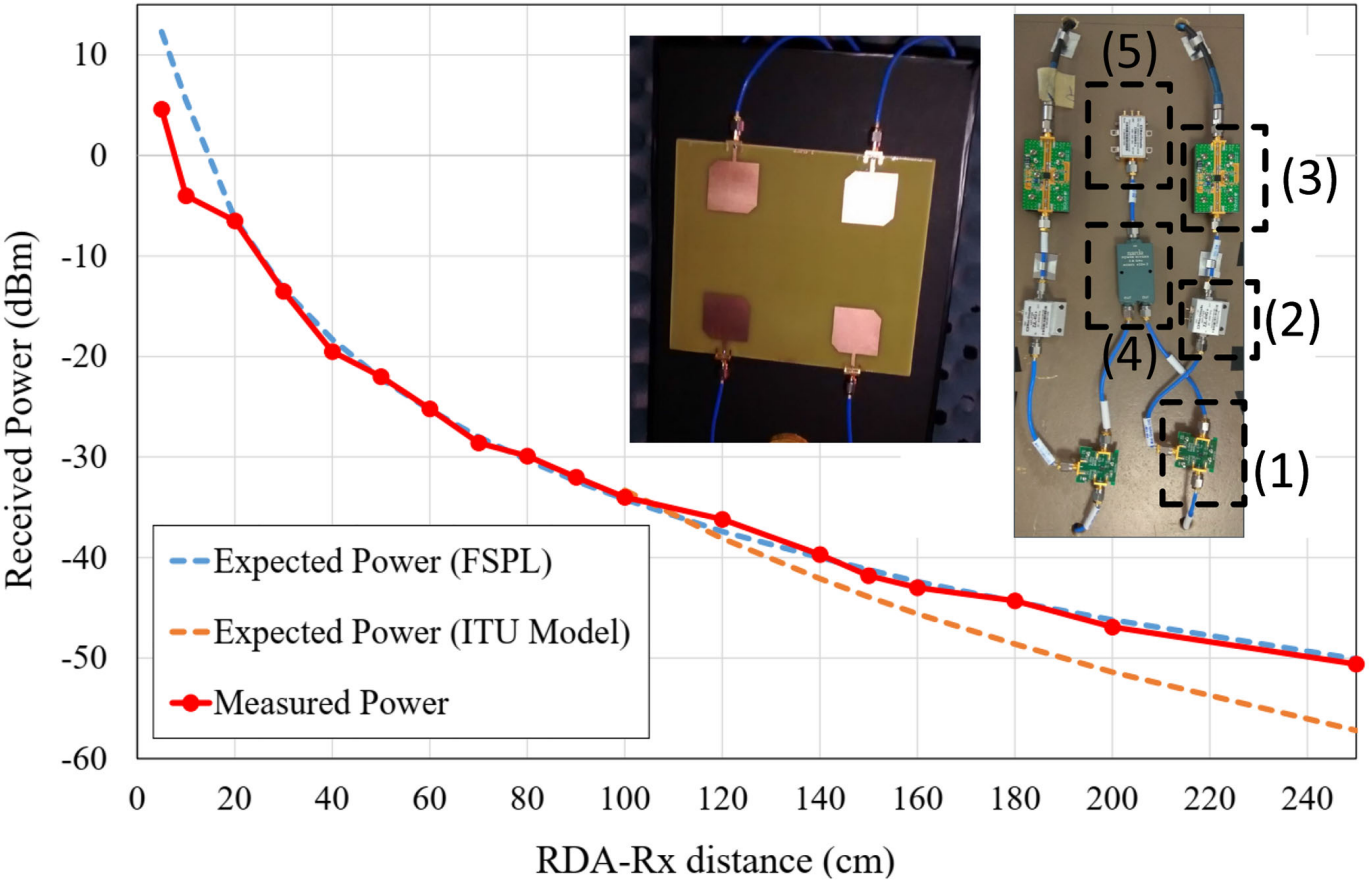
Measured received power at different distances from the RDA (broadside) in comparison with the theoretical expected power using the free-space path loss model (FSPL) and the indoor propagation ITU model [31]. The arrays for the dual-pol Pon RDA prototype (using CP antenna elements) are shown in the inset. RF components: including mixers (1), amplifiers (2 and 3), power dividers (4) and voltage control oscillators (5). CP antenna elements are employed to provide orientation flexibility for the OUS and the RDA.

To test the performance of the RDA system in the NF, an RDA system with lower gain and reduced cost when compared to [15], was designed with FR-4 material constituting a simple demonstrator as shown in Fig. 7 (see inset). The tracking plane is perpendicular to the RDA aperture containing the two-element arrays. Thus, each RF chain is defined by a receiving CP antenna element, a mixer (Linear Technology), a driver amplifier from (MiniCircuits ZFL-2500+), a power amplifier (TriQuint TQP9111), and a CP transmitting antenna.

Considering a two-element Pon-RDA, the received beacon signal at the antenna elements (i.e., $f_{RX}$) goes directly into the RF port of each mixer. It should be mentioned that all mixers share a common LO to ensure consistent reference. The output signal is then phase conjugated [12], thus an inverted progressive phase difference between antenna elements is achieved, resulting in a wavefront ($f_{TX}$) propagating towards the same direction as the beacon signal generated by the OUS. This process occurs in real-time, thereby performing analog signal processing [32].

The power received at broadside and at different distances from the OUS has been measured as shown in Fig. 7. The following NF expression for the path loss is considered [33]:

\begin{align*}
P(r,f) = \frac{G_{TX}G_{RX}}{4}\left(\frac{1}{(kr)^2}-\frac{1}{(kr)^4}+\frac{1}{(kr)^6}\right) {\kern-3.1298pt}, \tag{7}
\end{align*}

where $G_{TX}$ is the gain of the transmitter, $G_{RX}$ is the gain in reception, $k$ is the free-space wavenumber. The measured received power well approximates the trend for the free-space path loss model (Fig. 7). However, there is poor agreement with the indoor propagation model after about 1 m [31] which takes into consideration multipath effects. Such disagreement is justified in the fact that the measurements were made inside an anechoic chamber, replicating a free-space environment.

**Fig. 8.**
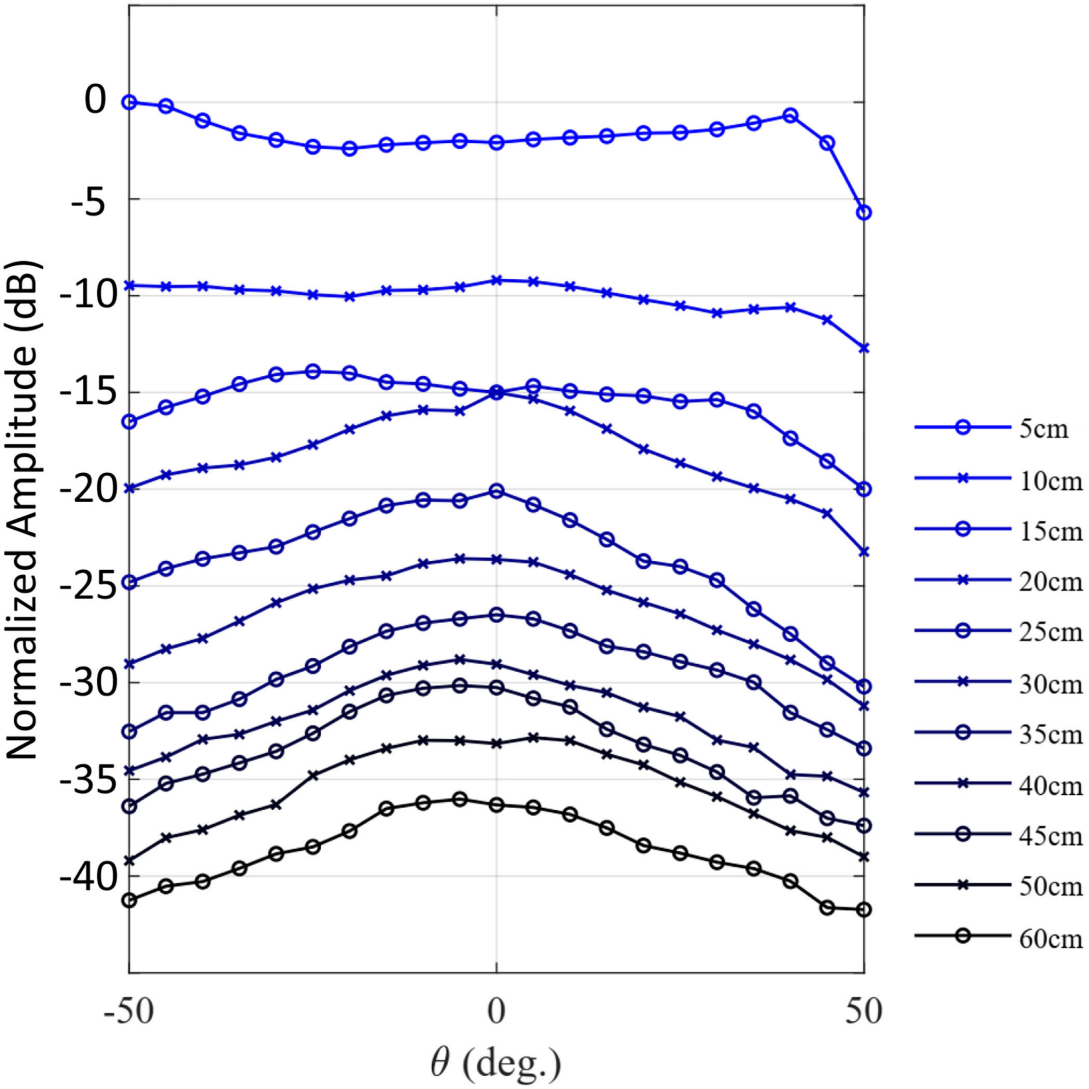
Monostatic measurements for various ranges; i.e. RDA to the OUS.

**Fig. 9.**
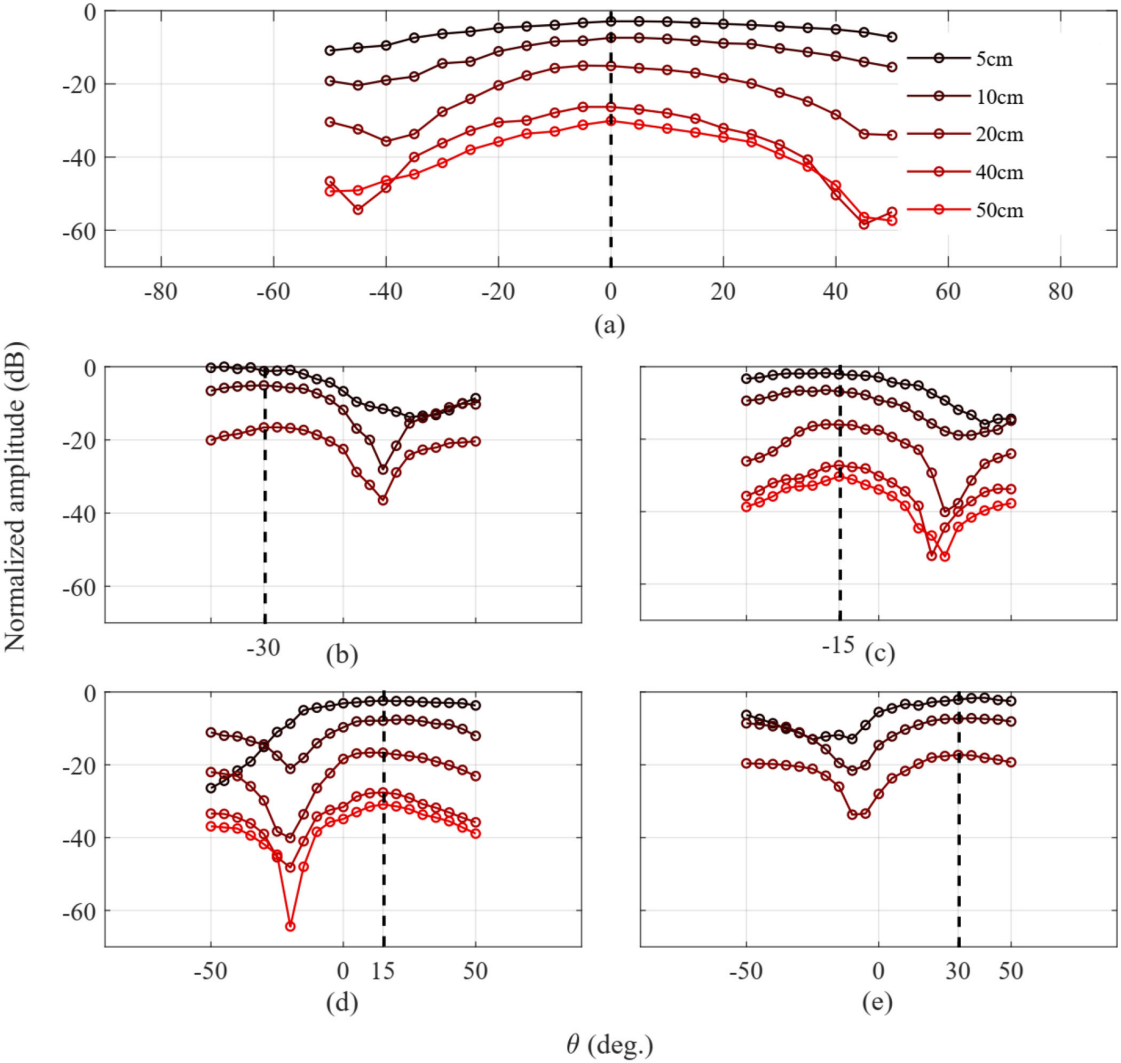
Bistatic measurements in the NF at different angles and distances for the proposed RDA: (a) 0 $^{\circ }$, (b) −30 $^{\circ }$, (c) −15 $^{\circ }$, (d) 15 $^{\circ }$ and (e) 30 $^{\circ }$.

**Fig. 10.**
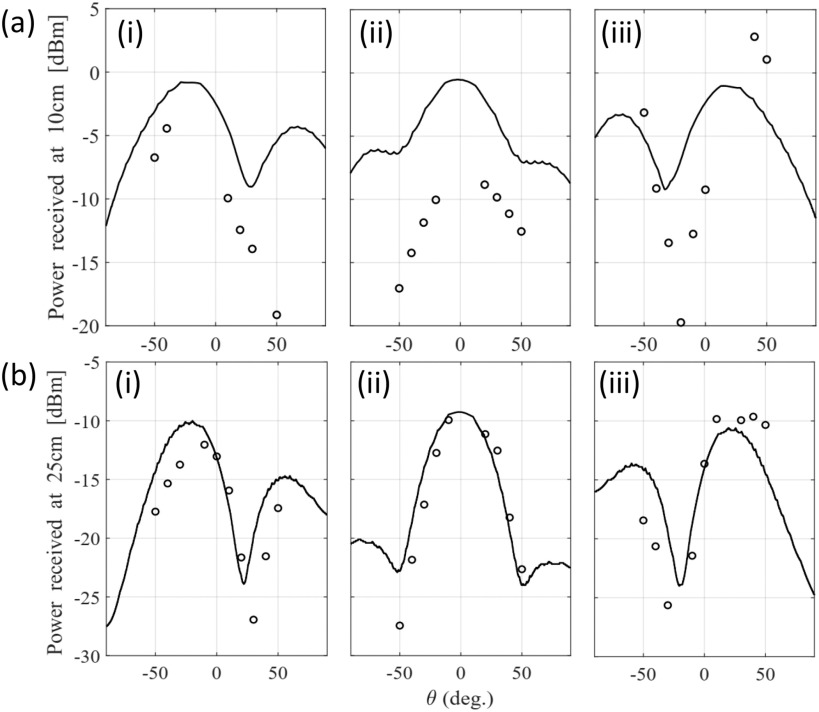
Bistatic measurements (circles) and simulations (cont. lines) for different ranges and angles: (a) 10 cm and (b) 25 cm, (i) −20 $^{\circ }$, (ii) 0 $^{\circ }$ and (iii) 20 $^{\circ }$.

Monostatic and bistatic measurements were completed for proof of concept (see Figs. 8 to 10). The procedure to acquire monostatic and bistatic patterns is illustrated in Fig. 7 of [15]. The bistatic pattern corresponds to the radiation pattern of the RDA when the beacon tone is set at a given angle. To test the tracking capabilities of the RDA, several bistatic patterns at different angular positions of the beacon signal are considered. The transmit signal of the OUS, i.e., the beacon, is kept at a given angle, while the receiver patch (which samples the field) rotates around to map the complete beam pattern for a given angular range. Therefore it is expected that the RDA will be continuously pointing and tracking its maximum back towards the beacon OUS.

Results of the monostatic measurements can be found in Fig. 8. A comparison of the normalized received power of the patch antenna for a range from −50 $^{\circ }$ to 50 $^{\circ }$ and of various distances from NF to FF is presented. The boundary in this case is at 35 cm. Respectively, results of the bistatic measurements can be found in Fig. 9. Comparison of the normalized received power for a range from −50 $^{\circ }$ to 50 $^{\circ }$ and of various distances from NF to FF, as well, for five given angles $\theta$: (a) 0 $^{\circ }$, (b) −30 $^{\circ }$, (c) −15 $^{\circ }$, (d) 15 $^{\circ }$ and (e) 30 $^{\circ }$.

Bistatic measurements at 10 cm and 25 cm have also been made and compared in this case to the simulations in Fig. 10. In Fig. 10(a) it can be observed that measurements and simulations have a similar field pattern. Despite the difficulties to make the measurements with high accuracy (the mechanical supports were very close together for the RDA and patch receiver), the shape of the measured points follows the expected trend. On the other hand, in Fig. 10(b), the measured points match very well to that of the expected pattern shape, also demonstrating that the required NF traking is possible.

### B. Health and Safety Discussion

The International Commission on Non-Ionizing Radiation Protection (ICNIRP) has set limitations on exposure to electromagnetic fields. There are two exposure scenarios, one for the occupational and one for the general public. Those two have different limits, in three categories. The first one refers to the whole body exposure and the others to the local head/torso and the local limb exposures, respectively. There are a number of different ways the exposure can be measured, the most common being the specific energy absorption rate (SAR, W/Kg). Table II shows the exposure limits for the five aforementioned categories, from 100 kHz to 6 GHz.

**TABLE II:** Electromagnetic Field Exposure Restrictions Limits, Based on ICNIR, From 100 kHz to 300 GHz [34]

Exposure	Whole-body av.	Local Head/Torso	Local Limb
scenario	SAR W/Kg	SAR W/Kg	SAR W/Kg
Occupational	0.4	10	20
General Public	0.08	2	4

The restrictions refer to average intervals of 30 minutes in terms of the whole-body average SAR and 6 minutes for the local SAR [34].

## IV. Virus Deactivation Through Microwave Radiation Experiments

This section describes the microwave heat experiment for coronavirus strain CoV-229E-GFP deactivation. The experiment demonstrated the protein denaturation and coronavirus deactivation, using a microwave open ended cavity system, which induced microwave heating of aqueous films.

### A. Inactivation of the Virus

The microwave open ended cavity system used for the heat experiments was originally developed to enable rapid bonding of individual microelectronic components on a board assembly [17], [35], [36]. This system has an advantage of volumetric and uniform heating, selective energy deposition with reduced processing times and increased energy efficiency. Microwave heating was achieved using this open-ended single mode resonant microwave applicator/oven which has been described in [35]. Test specimens consisted of 200 ${\mu }$l of Cov-229E-GFP diluted to a multiplicity of infection (MOI) of 0.3 in DMEM, in a cuvette of internal volume 8 mm × 8 mm × 8 mm. The samples were then heated at a constant ramp up rate of 1.5 $^{\circ }$C/s to the chosen inactivation temperature (50, 60, 70 and 100 $^{\circ }$C) held for varying amounts of time (10 seconds to 60 seconds). Residual virus infectivity was determined by infection of virus-susceptible Huh7 cells and measurement of virus replication growth curves as a function of the GFP fluorescence.

**Fig. 11.**
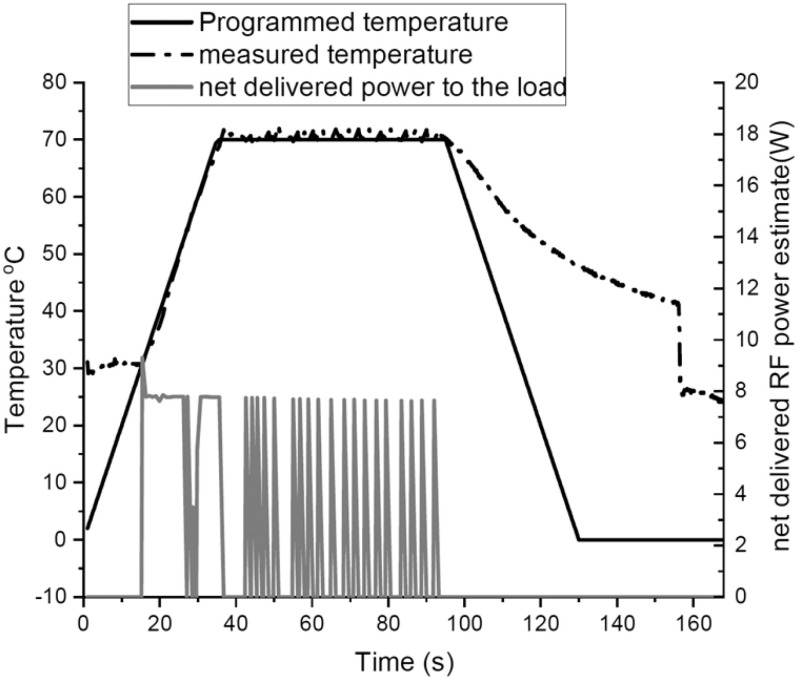
Net microwave power along with the temperature profile for a 70 $^{\circ }$C heating cycle experiment with the virus suspension for 60 s hold time period.

**Fig. 12.**
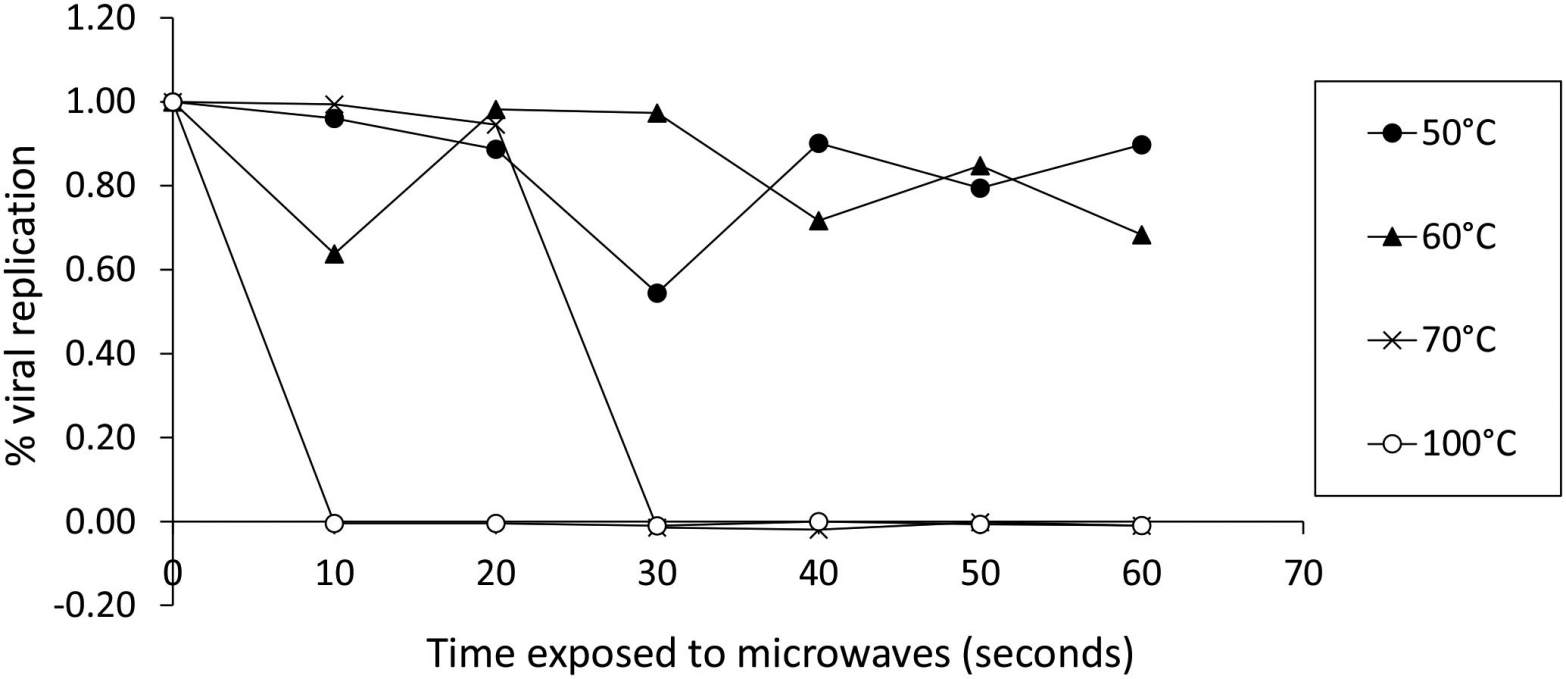
Survival rates of virus when exposed to the microwaves in an open ended microwave cavity for 10-60 s at four different temperatures: 50 °C, 60 °C, 70 °C and 100 $^{\circ }$C.

Fig. 11 shows the set and measured temperature profiles and net input power for a 70 $^{\circ }$C temperature cycle with 60 seconds set hold time. The net input microwave energy for this temperature cycle is 164 Joules, approximately. Fig. 12 indicates the survival rates of virus at various temperatures and exposure durations. It can be seen that the virus survival decreases with both time and temperature with a maximum of 30% virus death at 60 $^{\circ }$C, and 100% virus death following exposure to 70 $^{\circ }$C for 30 seconds.

### B. Visual Thermal Assay Based on Protein Denaturation

**Fig. 13.**
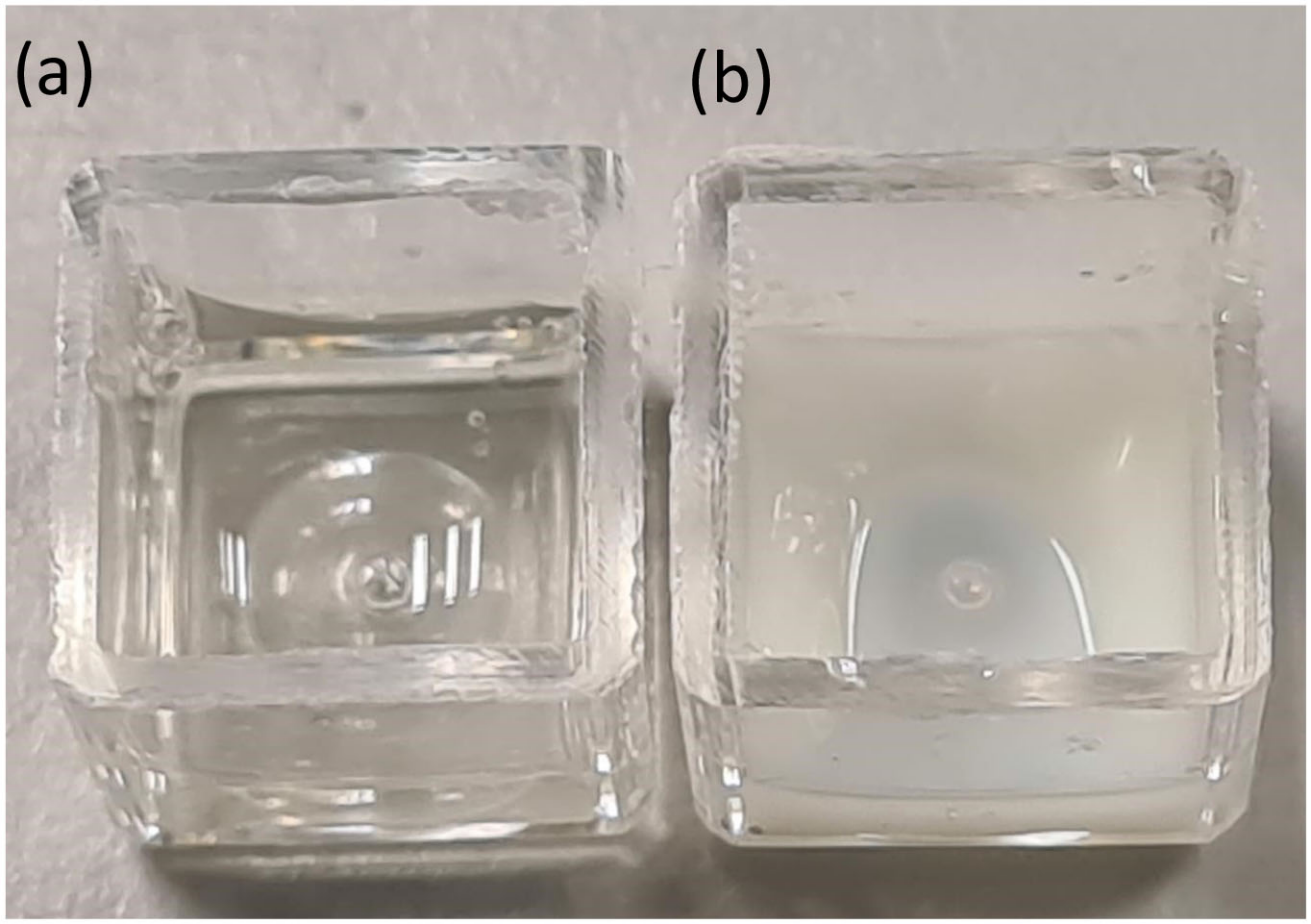
Microwave heated protein solution result to 60 $^{\circ }$C by microwave irradiation. (a) Colourless solution before being thermally heated. (b) White solution after thermal heating. The white colour in the solution shows that the protein has been denatured and gelled.

In order to demonstrate that microwave-induced heating of aqueous films can lead to protein denaturation and virus deactivation, an open microwave cavity as described above is used to irradiate protein solution (1:1 mixture of hen egg white and phosphate buffered saline (PBS) at pH 7.4). The protein solution was monitored for 2 minutes where there was an obvious colour change from colourless to white. The transparent solution turns white opaque upon irradiation, therefore giving a direct visual indication that the protein denatured and gelled. Hen egg white proteins are known to denature at 60 $^{\circ }$C [37]. This was also confirmed by another controlled experiment in which the egg white/PBS solution was thermally heated to 60 $^{\circ }$C. To this end, approximately 500 ${\mu }$L of the solution was pipetted onto a glass microscope slide. A second slide was placed on top of the solution to minimise loss of solution by evaporation. This was then placed on a variable temperature hotplate set to 60 $^{\circ }$C. This experiment was repeated with the open ended cavity system with a heating ramp up from 25 to 60 $^{\circ }$C at 1 $^{\circ }$C/s and a hold time of 60 seconds. In this second experiment, the solution was monitored for 2 minutes where there was an obvious colour change from colourless to white (Fig. 13) similar to the hotplate experiment.

While these three sets of experiments show that microwave irradiation of thin aqueous films to above 60 or 70 $^{\circ }$C can lead to protein denaturation and virus deactivation, it has to be emphasized that these experiments were done with a simpler microwave set-up as the one outlined in this publication, because the current RDA prototype does not yet have a powerful enough power source and beam directivity to deliver the required radiation intensities. Nevertheless, the experiments show that microwave heating to a modest 60 $^{\circ }$C is sufficient to achieve the desired damage to proteins and to viruses, respectively, and therefore to sterilize surfaces demonstrating proof-of-concept for the proposed methodology and approach for remote microwave sterilization. Two videos can also been found in the supplementary information about the hotplate and microwave cavity denaturing of egg white.

## V. Conclusion

A sterilization method applicable against the new coronavirus has been presented in this paper. The technique requires a small transmitter suitably positioned on the OUS to provide its location. The RDA receives the transmitted beacon signal and transmits power to the OUS until it reaches the desired temperature. A study has been done to test the tracking capabilities of the Pon RDA in the NF.

A liquid film of water was considered to produce heat for sterilization and the amount of power needed to deactivate the coronavirus was calculated. More specifically, the total time needed to reach 60 $^{\circ }$C. The studied liquid film has dimensions $\alpha$ = 14 cm, $b$ = 8 cm and thickness $t$ = 0.25 mm, whilst the total input power of the RDA is 3 W, maintaining below the exposure limits. The results showed that the study can be feasible for an azimuthal angle range from −40 $^{\circ }$ to 40 $^{\circ }$ and a distance $r$ = 10 cm. For $r$ = 25 cm the range reduced from −20 $^{\circ }$ to 20 $^{\circ }$. At the best case scenario (i.e., an OUS placed at broadside and $r$ = 10 cm) less than 4 minutes were needed to complete the required heating and the sterilization process. Better performances can be achieved indeed by increasing the power of the transmitter or increasing the aperture size of the RDA. Experiments have also been conducted to demonstrate that microwave-induced heating of aqueous films can lead to protein denaturation and virus deactivation. Measurements from these experiments show that the deactivation of viruses due to microwave induced heat is possible above 60 $^{\circ }$C and can be achieved with $< $ 99.9% certainty at 70 $^{\circ }$C above 20 seconds.

## Supplementary Materials

Supplementary materials
